# Effects of Different Pollination Treatments on the Appearance and Cell Development Characteristics of Blueberry Fruit

**DOI:** 10.3390/plants14213341

**Published:** 2025-10-31

**Authors:** Chunze Lu, Dian Liu, Jiayi Liu, Ke Li, Xinchun Wang, Jinying Li, Lin Wu, Ying Wang, Yanan Li

**Affiliations:** College of Horticulture, Jilin Agricultural University, Changchun 130118, China; lczjlau@163.com (C.L.); l25803617@163.com (D.L.); 15944070766@163.com (J.L.); l941515854@163.com (K.L.); m13455625075@163.com (X.W.); li_jy78@163.com (J.L.); linw@jlau.edu.cn (L.W.)

**Keywords:** Reka, blueberry, hybridization, fruit size, cell size, anatomic observe

## Abstract

The method of pollination significantly affects the growth and development of blueberry fruit. However, there remains a deficiency in systematic research regarding the impact of various pollination treatments on the cellular structure of blueberries. In the present study, a cross-pollination experiment was conducted on the blueberry varieties ‘Reka’, with the objective of comparing the characteristics of blueberry fruit cells under different pollination treatments. The results showed that different pollen sources had certain effects on the pedicel marks, calyx width and seed number of blueberry fruit. Simultaneously, various pollination treatments significantly affected both the transverse and longitudinal diameters as well as the individual fruit weight of the fruits. However, no significant differences were noted in the fruit shape index across the various pollination treatments. Within the internal cellular tissue of each treated fruit, certain indicators exhibited distinct variances. In the cell height of the inner region of the mesocarp, all cross-pollination treatments were significantly higher than self-pollination. The fruit of ‘Northland’ and ‘Blomidon’ were significantly lower than self-pollination in the number of cells in the outer area of the mesocarp. These effects lead to differences in cell arrangement and spatial distribution, resulting in differences in the correlation between cell indexes and fruit morphological indexes among different treatments. In the ‘Reka’ self-pollination treatment, the endocarp width was significantly negatively correlated with the fruit weight (*p* < 0.05), while the correlation in the other four treatments showed an upward trend, showing a positive correlation. This study comprehensively examined the formation of fruit cells under cross-pollination at the cellular level, thereby providing a cytological basis for understanding the development of blueberry fruit.

## 1. Introduction

Blueberry (*Vaccinium* spp.), a member of the Ericaceae family, is a small berry shrub producing fruits that are typically blue when ripe, though some varieties may exhibit red hues [1]. The fruit is known for its delightful taste, cherished by the public. According to a study released by the U.S. Department of Agriculture’s Human Nutrition Research Center, blueberries are among the most abundant sources of antioxidant nutrients, having been researched alongside more than 40 varieties of fruits and vegetables. The International Food and Agriculture Organization (FAO) recognizes blueberries as one of the top five foods beneficial to human health. As a result, the FAO has identified blueberries as one of the top five healthiest foods in the world [2]. Blueberries constitute a high-value crop with significant potential for development on a global scale [3].

Pollination serves as a vital ecological service in agriculture, facilitating fruit and seed production in approximately 70% of the world’s flowering plants [4]. The efficacy of pollination directly determines crop yield outcomes. Since Garfield’s initial identification of the Xenia [5] effect in apples, this phenomenon has been documented across diverse fruit tree species. Currently, it has been utilized to enhance both the yield and quality of fruit production, and the related research into the underlying mechanisms has progressively advanced [6]. Gupton’s [7] study demonstrated that pollen source variation significantly influences blueberry fruit maturation, providing early evidence of the Xenia effect in this crop. Nevertheless, the effectiveness of pollination is influenced by the compatibility among different varieties. In certain studies, the rate of fruit set resulting from self-pollination has been shown to surpass that of cross-pollination [8,9]. Empirical studies by Chavez et al. [10] and Gupton et al. [11] demonstrated that variations in mature seed count directly influence fruit set rate, seed number per berry, individual fruit weight and developmental period across multiple blueberry cultivars. Taber et al. [12] further highlight that cross-pollination of southern highbush blueberries enhance the fruit setting rate, fruit weight and seed number, while also reducing the time to fruit maturity. Numerous studies have indicated that cross-pollination typically improves fruit quality and is closely linked to the seed count per fruit; however, it is important to note that not all characteristics improve uniformly—in certain instances, an increase in fruit size may coincide with a decline in taste and flavor [8,13,14]. In addition to traits related to yield and appearance, the size and quantity of cells play a crucial role in determining both fruit size and yield potential [15]. For instance, Yang et al. [16] found a close correlation between the morphology of mesocarp cells and the appearance of fruits in European plums. Renaudin et al. [17] conducted a quantitative analysis of the temporal and spatial patterns of cell division and expansion in tomato peels. Wan et al. [18] systematically elucidated the alterations in cytological structure throughout the development of fruits and seeds of the blueberry varieties ‘Powderblue’, thereby providing a microscopic foundation for comprehending fruit development.

Pollination significantly impacts the development of blueberry fruit, as the morphogenesis of the fruit is fundamentally reliant on the proliferation and size of mesocarp cells. As a result, various sources of pollen may influence variations in fruit quality by altering cellular characteristics, which can lead to differing outcomes across distinct pollination treatments. Currently, there is a notable lack of systematic research concerning the effects of diverse pollination treatments on the cellular structure of blueberries. This study constitutes a comprehensive analysis at the cellular level used to examine the formation of pulp cells derived from the pollen of different male parent varieties on the fruit of the female parent, thereby providing a cytological foundation for understanding the developmental processes of blueberry fruit.

## 2. Results

### 2.1. Effects of Different Pollination Treatments on Blueberry Fruit

#### 2.1.1. Effects of Different Pollination Treatments on Fruit and Seed Development of Blueberry

**Figure 1:**
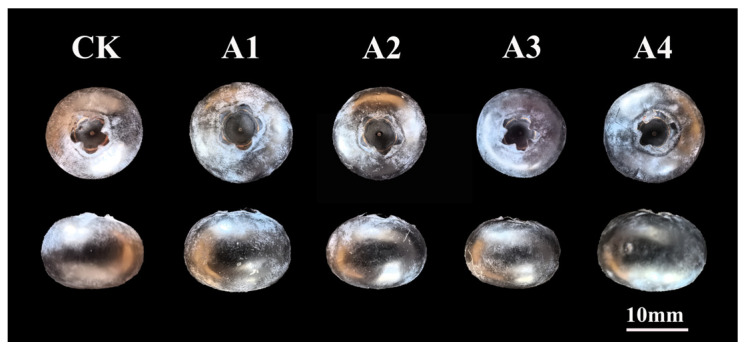
Morphological observation of blueberry fruit under different pollination treatments.

By observing the appearance of blueberry fruits under different pollination treatments (Figure 1), it was found that there were significant differences in fruit pedicel marks and calyx depressions between different pollination treatments. The fruit pedicel marks under A1 treatment exhibited significant differences when compared to those of the other four pollination treatments. The average diameter of the fruit pedicel marks exceeded 1.00 mm, thus achieving the intermediate standard, whereas the diameter of the fruit pedicel marks under the other treatments remained below 1.00 mm. The length of the fruit calyx depression under the CK, A3 and A4 treatments did not show significant differences and were greater than that observed under the A1 and A2 treatments. Furthermore, the number of single seeds per fruit in the A1, A2 and A4 pollination treatments were lower than that in the CK and A3 pollination treatments (Table 1). With regard to the percentage of fertile fruit, cross-pollination treatment resulted in a higher percentage than that of the CK treatment. It indicated that different blueberry pollen would influence the fruit and seed development.

#### 2.1.2. Effects of Different Pollination Treatments on the Size of Blueberry Fruit

**Figure 2:**
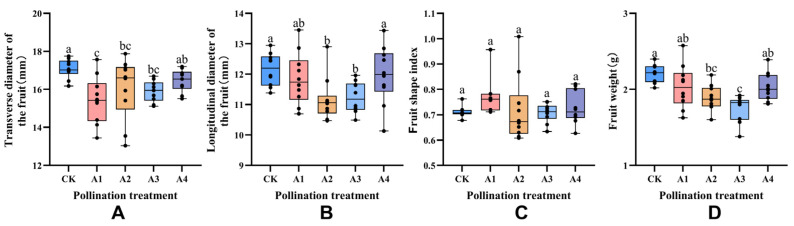
Effects of different pollination treatments on the size of blueberry fruit; different lowercase letters indicate significant differences under different treatments (*p* < 0.05). (**A**) is the Analysis of fruit transverse diameter under different pollination treatments. (**B**) Analysis of fruit longitudinal diameter under different pollination treatments. (**C**) Fruit shape index analysis under different pollination treatments. (**D**) Weight analysis of single fruit under different pollination treatments.

According to the size index of blueberry fruit under various pollination treatments (Figure 2), it was evident that different pollination treatments had distinct effects on the size of blueberry fruit. Significant differences were observed in the transverse diameter, longitudinal diameter and single fruit weight of blueberry fruit under different pollination treatments. Among them, A1 and A4 treatments generally tended towards the CK treatment, while A2 and A3 treatments were significantly lower than CK (*p* < 0.05). In the fruit diameter index, treatments A1, A2 and A3 were lower than the CK pollination group by 9.60%, 6.03% and 6.91%, respectively, while treatment A4 was comparable to CK. In terms of the fruit longitudinal diameter index, treatments A2 and A3 were significantly lower than CK (*p* < 0.05), at 8.07% and 7.49%, respectively. The A1 pollination group was lower than CK, but the difference was not significant, while treatment A4 was comparable to CK. Regarding the single fruit weight index, treatments A2 and A3 were 14.09% and 19.09% lower than CK, demonstrating a significant level (*p* < 0.05). Treatments A1 and A4 tended to align with the CK treatment.

### 2.2. Observation of Epidermal Cells Under Different Pollination Treatments

**Figure 3:**
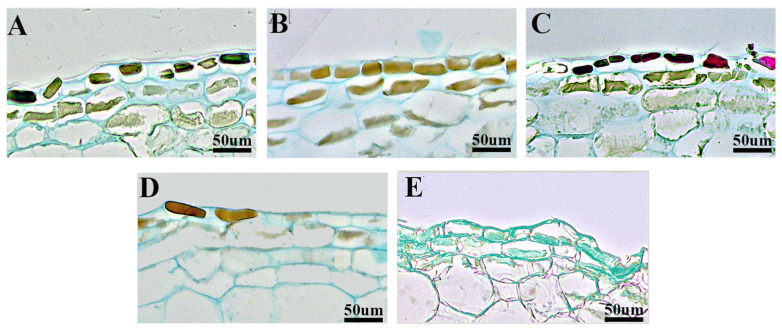
Observation of epidermal cells under different pollination treatments; (**A**–**E**) were epidermal cells treated with CK, A1, A2, A3 and A4, respectively. The images were taken in a 100× field of view.

**Figure 4:**
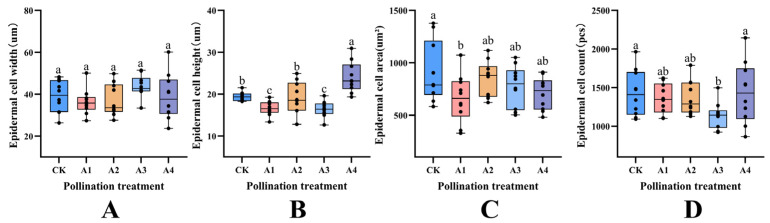
Effects of different pollination treatments on epidermal cell size of blueberry fruit. Different lowercase letters indicate significant differences under different treatments (*p* < 0.05); (**A**) analysis of endocarp cell width under different pollination treatments; (**B**) height analysis of endocarp cells under different pollination treatments; (**C**) analysis of single cell area of endocarp under different pollination treatments; (**D**) quantitative analysis of endocarp cells under different pollination treatments.

According to the observation of paraffin sections of fruits subjected to different pollination treatments (Figure 3), it was found that the fruit epidermis exhibited a consistent overall shape, with all specimens being flat rectangles that were closely arranged. Measurement and analysis of the transverse and longitudinal diameters of cells, as well as single cell area and cell number (Figure 4), revealed no significant difference in cell width among the various pollination treatments. However, the cell height of the A4 treatment was significantly higher than that of the other four treatments (*p* < 0.05). Specifically, treatments A1 and A3 recorded the lowest measurements, at only 69.29% and 68.21% of the A5 treatment, respectively. Additionally, the CK and A2 treatments were lower than A5 by 20.35% and 19.27%, respectively. The epidermal cells under treatments A1 and A4 tended to have similar single cell areas and were significantly lower than CK, but there was no significant difference in cell number when compared with CK.

### 2.3. Effects of Different Pollination Treatments on the Size of Mesocarp Cells in Blueberry Fruit

#### 2.3.1. Observation of Mesocarp Cells Under Different Pollination Treatments

**Figure 5:**
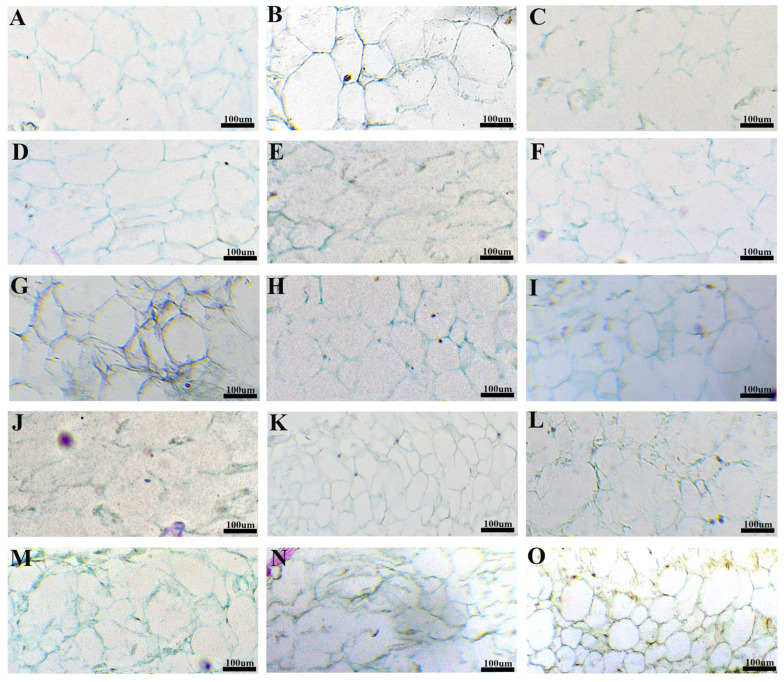
Observation of peel cells in blueberry under different pollination treatments; (**A**–**E**) are mesocarp outer cells CK, A1, A2, A3 and A4, respectively; (**F**–**J**) was the middle mesocarp cells treated with CK, A1, A2, A3 and A4, respectively. (**K**–**O**) was the inner cells of mesocarp under CK, A1, A2, A3 and A4 treatments, respectively. All images were taken in 40× field of view.

Through the observation of the mesocarp sections of the fruit (Figure 5), it was found that the shape of the outer cells of the mesocarp under A3 and A4 treatments was rectangular, which differed from that of the CK, A1 and A2 treatments (Figure 5A–E). There was no significant difference in the morphology of the middle cells of the mesocarp between A1, A2, A3 treatments and CK, and the cell dissolution was lighter, and the integrity of the cells was maintained. In contrast, the cell solubility under A4 treatment was high and resulted in most of the cells being broken and losing their integrity (Figure 5F–J). In terms of cell morphology in the mesocarp, the cells treated with A1, A2 and A3 largely lost their integrity due to dissolution and rupture, whereas the A4 treatment exhibited characteristics similar to CK, demonstrated only limited cell rupture and maintained cell integrity (Figure 5K–O).

#### 2.3.2. Effects of Different Pollination Treatments on the Size of Outer Mesocarp Cells

**Figure 6:**
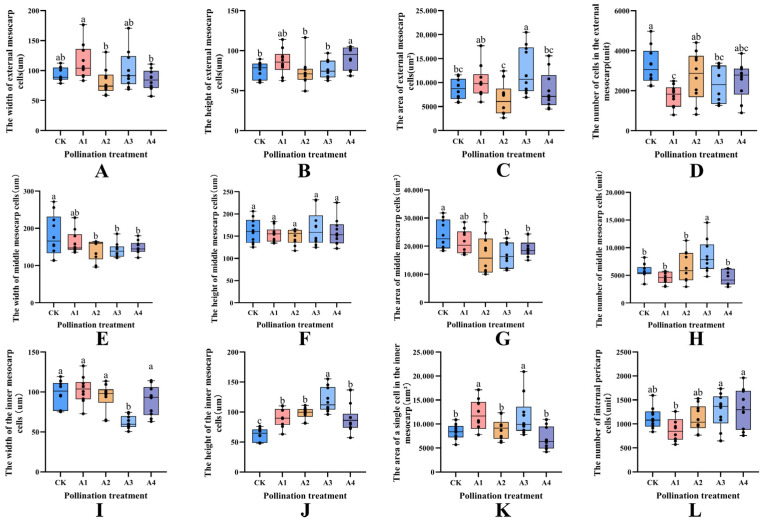
Effects of different pollination treatments on the size of mesocarp cells; different lowercase letters indicate significant differences under different treatments (*p* < 0.05). (**A**–**D**) were expressed as the analysis of mesocarp external width, height, single cell area and cell number, respectively. (**E**–**H**) was expressed as the analysis of the width, height, single cell area and number of cells in the middle of the mesocarp. (**I**–**L**) were expressed as the analysis of mesocarp internal width, height, single cell area and cell number, respectively.

According to the measurements and analyses conducted on the external cells of the mesocarp, there was no statistically significant difference in cell width observed between the CK treatment and treatments A1, A2, A3 and A4 (Figure 6A). However, it was noted that the A1 treatment exhibited a significantly greater width compared to treatments A2 and A4. Regarding cell height, a significant increase was observed in the A4 treatment in comparison to the CK treatment (*p* < 0.05), with an enhancement of 20.87%, while no significant differences were detected among the other treatments relative to the CK treatment (Figure 6B). Among the metrics of mesocarp external cells (Figure 6C), the single cell area of A3 treatment was the highest, measuring 12,509.84 ± 4968.16 um^2^, which is 49.34% greater than that of CK treatment. The A1, A2 and A4 treatments were comparable to the control group (CK), exhibiting no significant differences from CK. However, regarding the number of mesocarp external cells, the A1 and A3 treatments were similar and significantly lower than CK (*p* < 0.05), showing reductions of 46.38% and 28.5%, respectively (Figure 6D). There were no significant differences in the number of mesocarp external cells between the A2 and A4 pollination treatments and CK.

#### 2.3.3. Effects of Different Pollination Treatments on Cell Size in the Middle of Mesocarp

In the analysis of the middle cells of mesocarp (Figure 6E–H), the cell widths of the A2, A3 and A4 pollination treatments decreased by 21.91%, 21.85% and 17.61% relative to the control group (CK) and were significantly smaller than CK (*p* < 0.05) (Figure 6E). There was no significant difference between A1 and CK. The index of cell height did not exhibit a significant difference, with four pollination combinations trending toward the CK pollination group (Figure 6F). However, in the area of single cells (Figure 6G), the A2, A3 and A4 treatments decreased by 29.85%, 29.72% and 20.54% compared to CK and demonstrated a significant reduction relative to CK (*p* < 0.05). The A1 treatment was similar to CK, showing no significant difference. Regarding the number of cells (Figure 6H), the trends of A1, A2 and A4 treatments were consistent with CK, and there was no significant difference; however, A3 was significantly higher than CK (*p* < 0.05), increasing by 47.15% compared to CK during the same period.

#### 2.3.4. Effects of Different Pollination Treatments on Cell Size in Inner Mesocarp

Compared to the other three treatments during the same period, the A3 treatment exhibited a significantly lower width of mesocarp cells than CK (*p* < 0.05), with a decrease of 36.67%, while the other three treatments showed trends similar to CK (Figure 6I). In the internal cell height index of the mesocarp (Figure 6J), all cross-pollination treatments were higher than CK, with A3 being the highest, significantly exceeding CK by 91.67%. The following treatments were A1, A2 and A4, and they exhibited increases of 42.68%, 56.97% and 42.42%, respectively. Regarding single cell area, the A1 and A3 treatments had the highest values, significantly surpassing CK (*p* < 0.05), with increases of 43.03% and 39.02%, respectively (Figure 6K). There was no significant difference between the other two treatments and CK. In terms of cell count (Figure 6L), all cross-pollination treatments were similar to CK; however, the A3 and A4 treatments were significantly higher than A1 (*p* < 0.05), reached 1274.46 ± 353.58 and 1317.66 ± 406.34, respectively.

### 2.4. Effects of Different Pollination Treatments on Cell Size of Blueberry Endocarp

**Figure 7:**
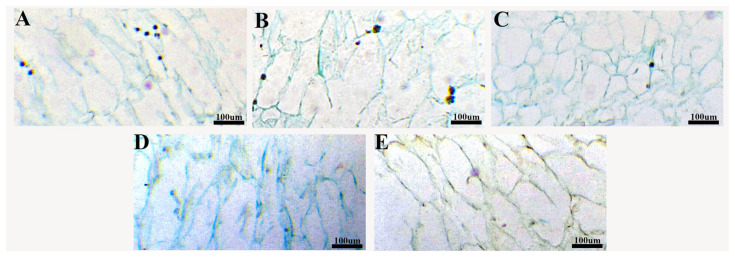
Observation of endocarp cells of blueberry fruit under different pollination treatments; (**A**–**E**) were endocarp cells treated with CK, A1, A2, A3 and A4, respectively. All images were taken in 40× field of view.

Compared to other treatments, the endocarp cells under A3 treatment were generally rounded (Figure 7). The endocarp of other treated blueberries appears slender overall. The cells treated with CK, A1 and A3 were not as closely arranged in structure compared to those treated with A2 and A4, due to cell dissolution.

**Figure 8:**
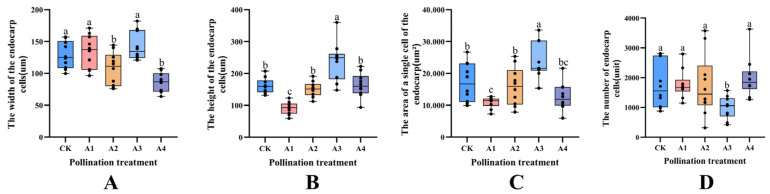
Effects of different pollination treatments on the size of endocarp cells; Different lowercase letters indicate significant differences under different treatments (*p* < 0.05). (**A**) is the analysis of endocarp cell width under different pollination treatments. (**B**) is the height analysis of endocarp cells under different pollination treatments. (**C**) is the analysis of single cell area of endocarp under different pollination treatments. (**D**) is the quantitative analysis of endocarp cells under different pollination treatments.

The cell width for treatments A2 and A4 was significantly reduced by 16.46% and 33.49%, respectively, when compared to the control group (CK) (*p* < 0.05). The cell height was like that of CK, showing no significant difference. Among the indicators of cell height, treatments A1 and A3 showed significant differences from CK (*p* < 0.05). Specifically, A1 exhibited a decrease of 43.17%, while A3 was significantly higher than CK by 48.22% (*p* < 0.05). Due to the variation in endocarp cell height, the trend in single cell area closely mirrored cell height. The performance of A1 was significantly lower than that of CK (*p* < 0.05), measuring 10,898.1 ± 1733.56 um^2^/unit, which is 36.66% lower than the CK treatment. Conversely, A3 treatment was 38.75% higher than that of the control group, reaching 23,872.11 ± 5672.29 um^2^/unit. There was no significant difference between the other treatments and the control (CK). In terms of cell count, only the A3 treatment was significantly different when compared to CK, showing a reduction of 43.31% compared to CK with the same index. The other three treatments were similar to CK, with an average of 1810.9825 ± 739.86 (Figure 8).

### 2.5. Effects of Different Pollination Treatments on the Correlation Between Blueberry Fruit Cells and Fruit Morphological Indexes

**Figure 9:**
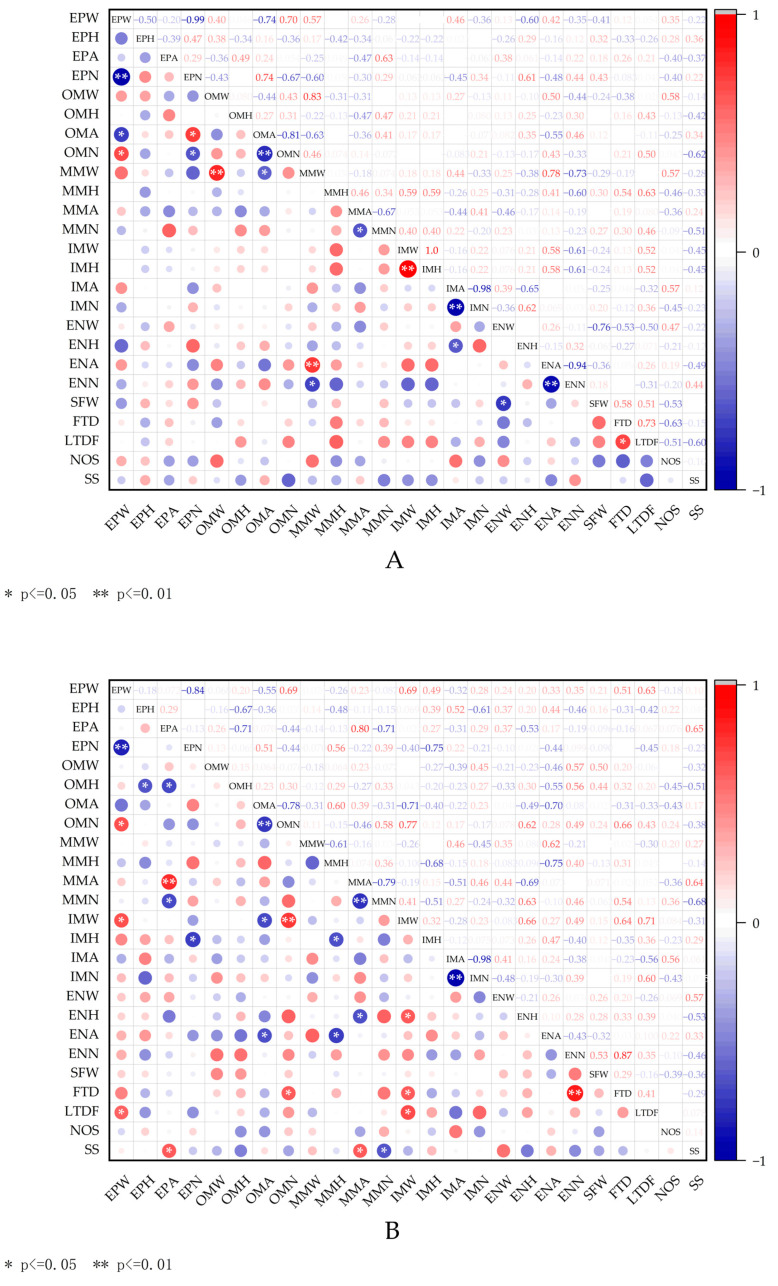

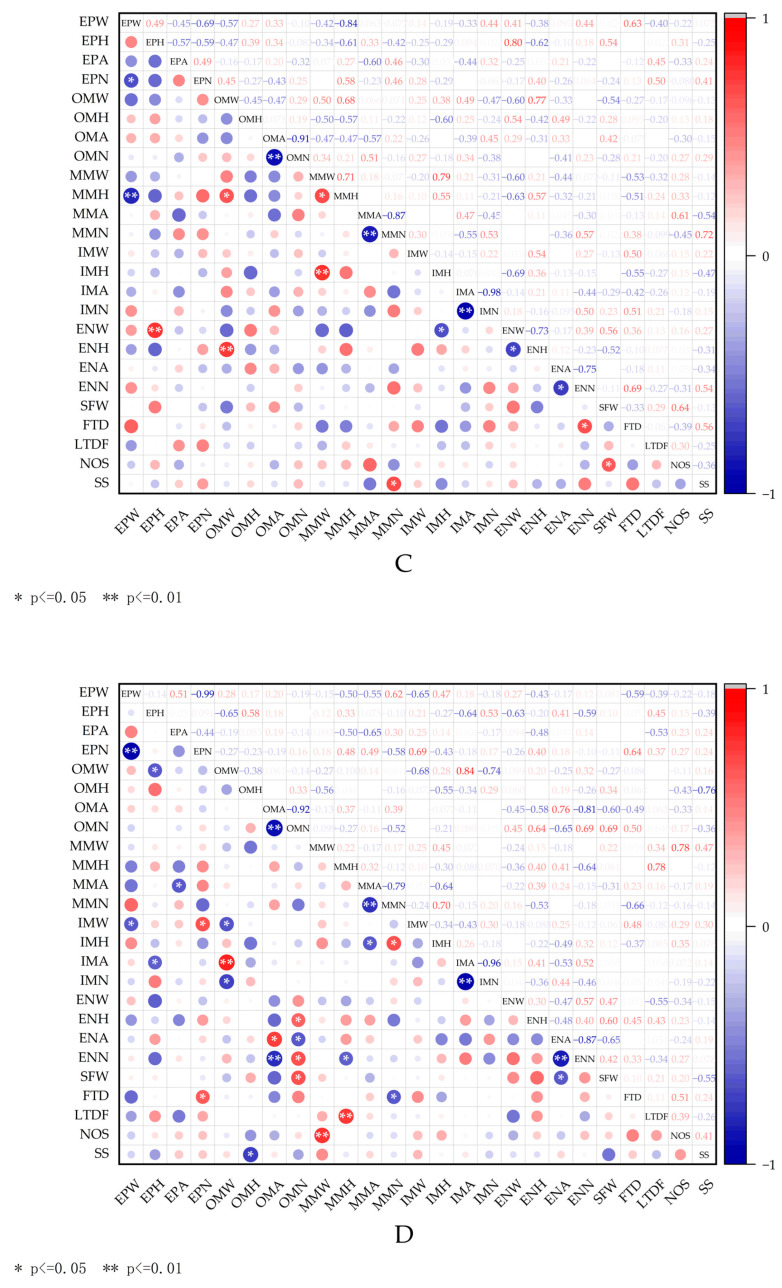

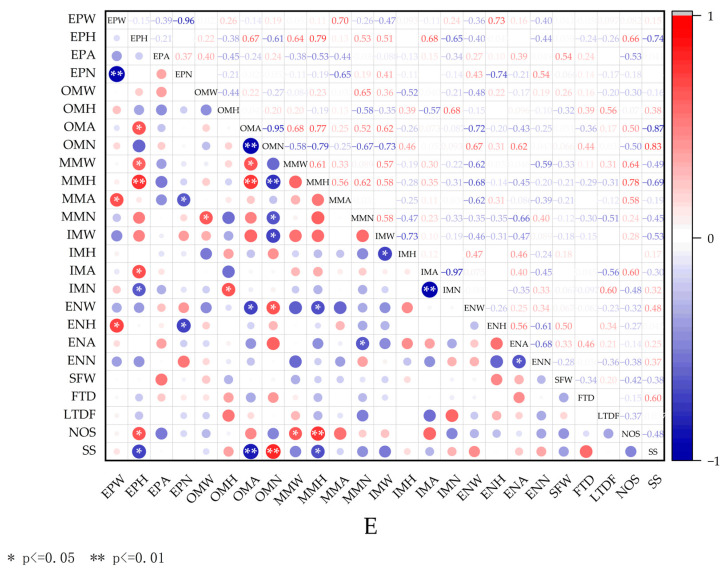
Heatmap of correlation between fruit cell size index and fruit morphological index under different pollination treatments. (**A**–**E**) in the figure represent the correlation analysis between fruit cells and fruit morphological indexes under CK, A1, A2, A3 and A4 pollination treatments, respectively. Among the indicators on the left side of the figure, EPW is the width of epidermal cells, EPH is the height of epidermal cells, EPA is the single area of epidermal cells, EPN is the number of epidermal cells, OMW is the width of outer cells of mesocarp, OMH is the height of outer cells of mesocarp, OMA is the single area of outer cells of mesocarp, OMN is the number of outer cells of mesocarp, MMW is the width of middle cells of mesocarp, MMH is the height of middle cells of mesocarp, MMA is the single area of middle cells of mesocarp, MMN is the number of middle cells of mesocarp, IMW is the width of inner cells of mesocarp, IMH is the height of inner cells of mesocarp, IMA is the area of inner cells of mesocarp, IMN is the number of inner cells of mesocarp and ENW is the width of inner cells of endocarp. In the indicators below the picture, SFW is the single fruit weight, FTD is the transverse diameter of the fruit, LTDF is the longitudinal diameter of the fruit, NOS is the number of seeds and SS is seed size. Close to 1 (red) indicates a strong positive correlation, close to −1 (blue) indicates a strong negative correlation and close to 0 (white) indicates no significant correlation. (*: *p* < 0.05, **: *p* < 0.01).

Through the correlation analysis of 25 indicators in fruits under different pollination combinations (Figure 9), it was found that the correlation between the number of epidermis and the width of epidermis was significantly negatively correlated with all pollination treatments except A2 treatment (*p* < 0.01), and A2 treatment also reached a significant negative correlation (*p* < 0.05). In the correlation between external mesocarp area and epidermal cells, only CK was significantly negatively correlated (*p* < 0.05), while A2, A3 and A4 treatments showed positive correlation. CK treatment showed a significant positive correlation between the number of external mesocarp cells and epidermal cells, and the correlation of all cross-pollination treatments decreased. Among them, A2 and A3 treatments reached a negative correlation, and the correlation was −0.43 and −0.19, respectively. Furthermore, a significant negative correlation was observed between the width of the endocarp and fruit weight in the CK treatment (*p* < 0.05), with a correlation coefficient of 0.76. In the remaining four treatments, the width of endocarp cells exhibited a positive correlation, with the largest positive correlation being 0.56 in the A2 treatment. Furthermore, in the correlation analysis between the number of mesocarp external cells and single fruit weight, the other four treatments in the group showed significant differences when compared to CK. The number of cells under the A1, A3 and A4 treatments was positively correlated with the single fruit weight, with A3 showing a significant positive correlation (*p* < 0.05), where the correlation coefficient reached 0.69. Conversely, the A2 treatment exhibited a negative correlation. In the A1 treatment, the number of external mesocarp cells, the width of internal mesocarp cells and the number of endocarp cells were significantly positively correlated with the transverse diameter of the fruit (*p* < 0.05), demonstrating a significant improvement compared to the CK treatment under the same index. At the same time, the correlation between the A2 and A3 treatments on the fruit’s transverse diameter under the same index was also improved compared to the CK treatment. A significant positive correlation was observed between the number of endocarp cells treated with A2 and the transverse diameter of the fruit (*p* < 0.05). Furthermore, the width of epidermal cells and the width of mesocarp cells under A2 treatment exhibited a significantly positive correlation with the longitudinal diameter of the fruit (*p* < 0.05), which was higher than that of the CK treatment under the same index. In contrast, the correlation of A4 treatment in this index was lower than that of CK, while the other two treatments displayed a clear negative correlation. In the A3 treatment, the cell height in the middle of the mesocarp was significantly positively correlated with the longitudinal diameter of the fruit (*p* < 0.05), which was consistent with the correlation observed in the CK treatment under the same index. The other three treatments in the group exhibited a lower correlation than CK in this regard, with A4 displaying a negative correlation of −0.31. The correlation between the number of seeds and the cell width in the middle of the mesocarp was positively correlated (0.57) in CK treatment, while the correlation decreased in A1 and A2 treatments, but it showed extremely significant positive correlation (*p* < 0.01) and significant positive correlation (*p* < 0.05) under A3 and A4 treatments, respectively. In the correlation between seed size and epidermal cell area, CK showed no significant negative correlation (−0.37), but it was significantly positively correlated in A1 treatment (*p* < 0.05), and A2 and A3 treatments also showed positive correlation.

## 3. Discussion

Fruit size is one of the important quality traits that directly impacts the yield of fruit. Studies have demonstrated that different pollen sources significantly influence the size of blueberry fruit [19]. In this experiment, it was found that the pollination combination of ‘Reka’ × ‘Northland’ had the most pronounced effect on pedicel size, calyx width and seed number. Various pollination treatments exhibited significant effects on both the transverse and longitudinal diameters, as well as the single fruit weight. Notably, the fruit transverse diameter was greatest under the self-pollination of ‘Reka’ and the pollination combination of ‘Reka’ × ‘Toro’. The trend of fruit longitudinal diameter and single fruit weight was the same, and they all performed best in the three pollination combinations of ‘ Reka ‘ selfing, ‘ Reka ‘ × ‘ Northland ‘ and ‘ Reka ‘×’ Toro ‘. The results presented above indicate that significant differences exist in the effects of various pollination combinations on fruit morphological indices, which is consistent with the research conducted by Nagasaka et al. [20]. This may be attributable to the distinct genetic characteristics of the male parent varieties. The genome associated with pollen exerts a regulatory influence on fruit morphogenesis. Although the different pollination treatments resulted in notable differences in the appearance of blueberry fruit, the calculations for the fruit shape index revealed that the pollen source did not have a significant impact on the fruit shape index. This suggests that different treatments can regulate fruit size, while they do not alter the fundamental shape of the fruit. Additionally, the number of seeds within the fruit varied depending on the influence of different pollen sources. The impact of different pollen sources on the seed count in the fruit aligns with the viewpoint of Ehlenfeldt [8], Gupton [7] and Miller et al. [14] that the pollen from large-fruit blueberry varieties may enhance the fruit enlargement of the female parent. Concurrently, various pollination treatments also had a notable effect on the diameter of the fruit pedicle marks of blueberry fruit, which may be related to the physiological activity of the pollen and the mechanisms of nutrient distribution during fruit development.

The aforementioned differences in appearance must have an underlying cytological basis. Fruit development commences with the division and expansion of cells following pollination and fertilization [21]. The final size of the fruit is determined by the number and volume of cells [15,22]. To investigate the mechanism behind the phenotypic differences observed in this experiment, the cell size structures of blueberry fruits were microscopically examined and analyzed using the paraffin section method. The results indicated that there were significant differences in cell size, number and morphology across various tissues (epidermis, outer region of mesocarp, middle region of mesocarp, inner region of mesocarp and endocarp) in blueberry fruits subjected to different pollination treatments. Regarding cell size, the central region of the mesocarp was most significantly affected by pollen sources, followed by the cells in the inner region of the mesocarp and the endocarp; in contrast, the area of the outer mesocarp and epidermal cells demonstrated less sensitivity to pollen sources. The cell area of ‘Northblue’, ‘Madion’ and ‘Toro’ was significantly smaller than that of ‘Reka’. Notably, the fruit cells derived from ‘Madion’ as the male parent were smaller and exhibited a denser arrangement, which may contribute to enhancing the hardness of the fruit [23], ultimately influencing storage and transportation [24]. On the other hand, changes in cell morphology may also affect the structure of the cell wall and further impact the texture of the fruit [13,25]. In addition, Yang et al. [13] researched six European plum varieties and found that different pollination combinations significantly influence the development of stone cells in their fruits. Similarly, the development of stone cells in blueberry fruit may also be influenced by different pollen sources, thereby affecting the texture and taste of the fruit [26,27]. Cell division determines the total number of cells in the fruit, and an increase in the total number of cells directly promotes the growth of fruit volume and weight. This experiment demonstrated that there were significant differences in the effects of various pollination treatments on the number of fruit cells. For instance, the number of peel cells in different parts of the fruit of the semi-highbush blueberry variety ‘Northland’, when used as the pollination source, was significantly lower than that of the ‘Reka’ self-pollination treatment. The ‘Madion’ pollen resulted in a significantly lower number of epidermal cells, mesocarp external cells and endocarp cells when compared to self-pollination, whereas the number of cells in the middle of the mesocarp was higher than that of ‘Reka’ self-pollination. This may be related to the hormone levels and nutrient supply found in the pollen. Simultaneously, the changes in cells also led to changes in the correlation between the indicators under different pollination treatments. To illustrate, in the correlation between external mesocarp area and epidermal cells, all cross-pollination treatments were higher than the correlation under self-pollination treatment. The endocarp width of fruits that were self-pollinated with ‘Reka’ exhibited a significantly negative correlation with fruit weight (0.76), whereas the endocarp cell width of fruits subject to the other four cross-pollination treatments displayed a positive correlation. The correlation in treatments involving ‘Northblue’ and ‘Madion’ as male parents reached 0.56 and 0.47, respectively (Figure 9). This research elucidated the impacts of various pollen sources on the quality formation of blueberry fruits from a cytological perspective, thereby offering a theoretical foundation for the selection of pollination varieties and the regulation of fruit quality, as well as providing a new insight into the physiological mechanisms underlying fruit development.

## 4. Materials and Methods

### 4.1. Test Sites and Plant Materials

**Figure 10:**
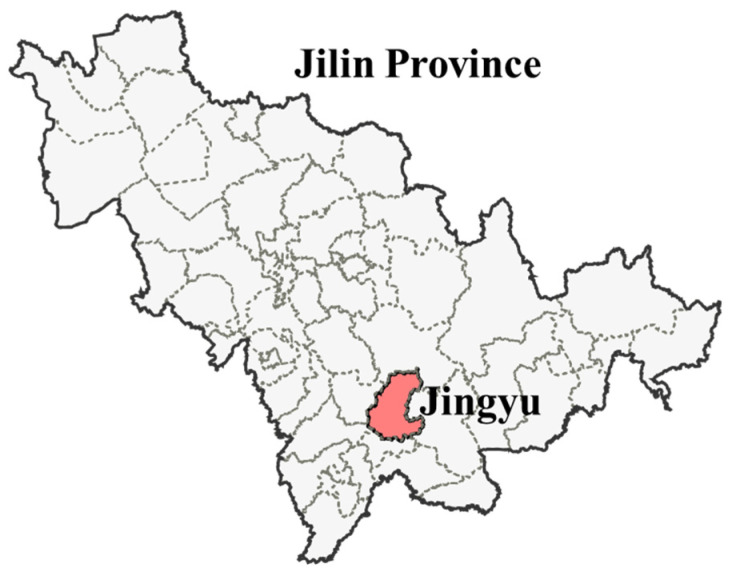
The geographical distribution map of the test site.

The test site is situated in the Jingyu Blueberry Science and Technology Courtyard (Small Berry Cultivation Base), located in Yishenchang Village, Huayuankou Town, Jingyu County, Baishan City, Jilin Province (Figure 10). Its coordinates are 42°47′24′’ N latitude and 127°8′41′’ E longitude, at an altitude of 447 m. The average annual temperature at this location is 3.7 °C, with an average annual precipitation of 776.4 mm. The frost-free period extends for approximately 130 days, and the soil composition is characterized as slightly acidic black loam. The adult blueberry varieties ‘Reka’ (7 years old), which has been maintained under identical management conditions, was selected as the female parent plant. The pollens of the cultivars ‘Northland’, ‘Northblue’, ‘Blomidon’ and ‘Toro’ were utilized as the pollen donor.

### 4.2. Pollen Collection and Pollination

In this study, the ‘Reka [28]’ variety was selected as the female parent, while ‘Northland [29,30]’, ‘Northblue [29,30]’, ‘Blomidon [31]’ and ‘Toro [32]’ varieties were utilized as male parents for hybrid pollination. The self-pollination of blueberry variety ‘Reka’ served as the control group, denoted as CK. Cross-pollination combinations including ‘Reka’ × ‘Northland’, ‘Reka’ × ‘Northblue’, ‘Reka’ × ‘Blomidon’ and ‘Reka’ × ‘Toro’ were labeled as pollination treatments A1, A2, A3 and A4, respectively (among the cross-pollinated varieties, except for the variety Blomidon, which bloomed before the flowering of the mother plant, the other three varieties coincided with the flowering of the mother plant). Anthers from selected pollinizers were collected from the Jilin Jingyu Blueberry Science and Technology Courtyard, a small berry cultivation facility. Before the beginning of pollination, the mature buds to be opened on the day of selecting the male parent variety were brought back to the laboratory and dried under natural conditions. After the petals were expanded and the anthers were cut, the pollen was prepared and stored in the refrigerator at 4 °C. Three female parent plants with the same management level and the same growth status were randomly selected in the experimental site. In the course of the experiment, each treatment selected 3~4 inflorescences in different directions on the same mother plant. Each inflorescence was left with three buds that were about to open on the day (about 1 day before flowering), followed by emasculation and pollination, and then immediately set in a pollination bag and tagged (one female parent plant was repeated once, and there were three replicates in the experiment. Each pollination treatment pollinated about 30 plants in total). The pollination bag was removed 6 days after pollination.

### 4.3. Study on the Blueberry Fruit Development Under Different Pollination Treatments

Upon maturation of the pollinated fruit, measurements were taken of the transverse diameter, longitudinal diameter and the scar diameter at the base of the berry using a vernier caliper. Additionally, the width and depth of the fruit calyx were observed. The fruit shape index (FSI) was calculated as the ratio of longitudinal to transverse diameter. Fruit shape was classified according to Han’s [18] fruit shape standards: oblate (0.6~0.8), round to nearly round (>0.8~0.9), oval to conical (>0.9~1.0) and slender (>1.0). Scar at the base of berry size was categorized based on the blueberry germplasm resource description spectrum: small (<1.0 mm), medium (1.0~1.5 mm) and large (>1.5 mm). Individual fruit fresh weight was quantified using a precision analytical balance, and photographs were captured to document the alterations in fruit resulting from various pollination treatments.

### 4.4. Study on the Cell Characterization of Lingonberry Fruit Under Different Pollination Treatments

Cellular characteristics of blueberry fruits were analyzed using standard paraffin sectioning techniques. Mature ‘Reka’ blueberries from distinct pollination treatments were harvested and fixed in FAA solution under vacuum infiltration (the three fruits were used for one experimental repetition, and this repetition was carried out three times in total), followed by 4 °C refrigeration. Specimens were transversely sectioned into 3–5 mm slices and subjected to graded ethanol dehydration (70%—85%—95%—100%), each level of dehydration lasting 1–2 h. Subsequently, the samples were sequentially cleared in ethanol-xylene mixtures (1:1) and pure xylene. Each sample was soaked in xylene for one hour. Tissue embedding was performed in paraffin at 58–60 °C. Serial sections were prepared with a Leica RM 2235 microtome with a slice thickness of 10 um, dual-stained with safranin O and fast green and mounted with neutral balsam. Imaging and morphological analysis were conducted using an OLYMPUS BX53 LED microscope.

Cellular morphometrics across five blueberry fruit tissues were quantified using ImageJ 1.45f software, measuring cell width (periclinal cell diameter), height (anticlinal cell diameter) [17] and cross-sectional area (epidermis, outer mesocarp, middle mesocarp, inner mesocarp and endocarp). The changes in cell number and cell size after ripening of blueberry fruit under different treatments were analyzed through Wan’s [18] quantitative method. Excel 2016 and Origin 2022 software were used for data analysis and plotting.

## 5. Conclusions

Different pollination treatments significantly influenced the pedicel marks, calyx width and seed number of blueberry fruit, with the ‘Reka’ × ‘Northland’ pollination combination exhibiting the greatest impact. Furthermore, the pollen source had a significant effect on the transverse diameter, longitudinal diameter and single fruit weight of the fruit; however, it did not alter the fruit shape index. Cytological observations indicated that the pollen source had a significant effect on the morphological and quantitative indices of most pulp cells, except for certain parameters of the epidermis and some mesocarp cells. These differences at the cellular level altered the arrangement and spatial distribution of cells, which subsequently resulted in significant variations in the correlation between intercellular indexes and morphological indexes, such as the transverse and longitudinal diameter of the fruit, as well as the weight of individual fruits, due to the use of different pollen sources. This study systematically elucidated the role of pollen xenia in the development of blueberry fruit at both morphological and cytological levels and provided a crucial theoretical foundation for enhancing fruit quality by strategically selecting optimal pollination trees in practical production.

## Figures and Tables

**Table 1:** Effects of different pollination treatments on blueberry fruit and its seeds.

Pollinating Treatments	Berry Bloom	Scar at the Base of Berry Size	Berry Status of Calyx	Berry Depth of Eye Basin	Berry Width of Eye Basin	Number of Seed	Seed Size	Percentage of Fertile Fruit (%)
CK	**	-	**	*	**	*	**	76.09 d
A1	**	*	**	*	*	-	**	82.44 c
A2	**	-	**	*	*	-	**	82.95 c
A3	**	-	**	*	**	*	**	90.67 a
A4	**	-	**	*	**	-	**	84.4 b

Note: In the table, Berry bloom: - for no, * for thin, ** for thick; scar at the base of berry size: - is small, * is medium, ** is large; berry status of calyx: - for shedding, * for residual, ** for persistent; berry depth of eye basin: - flat, * shallow, ** deep; berry width of eye basin: - for narrow, * for medium, ** for wide; number of seed: - is less, * is medium, ** is more; seed size: - is small, * is medium, ** is large. As indicated by the letters “a”, “b”, “c” and “d”, there are statistically significant differences between treatments at the 0.05 *p*-value cutoff.

## Data Availability

The original contributions presented in this study are included in the article. Further inquiries can be directed to the corresponding author.
